# Discovery of novel highly active and stable aspartate dehydrogenases

**DOI:** 10.1038/s41598-017-05522-7

**Published:** 2017-08-11

**Authors:** Hao Li, Taicheng Zhu, Liangtian Miao, Dan Zhang, Yongxian Li, Qi Li, Yin Li

**Affiliations:** 1 Key Laboratory of Industrial Biotechnology, Ministry of Education, Jiangnan University, Wuxi, 214122 JiangSu Province P.R. China; 2 CAS Key Laboratory of Microbial Physiological and Metabolic Engineering, Institute of Microbiology, Chinese Academy of Sciences, Beijing, 100101 P.R. China

## Abstract

Aspartate family amino acids (AFAAs) have important commercial values due to their wide spectrum of applications. Most if not all AFAAs are produced under aerobic conditions which is energy-intensive. To establish a cost-effective anaerobic process for production of AFAAs, it holds great promise to develop a new pathway enabling the conversion of oxoloacetate into aspartate through direct amination which is catalyzed by aspartate dehydrogenase (AspDH). Compared with the well studied aspartate aminotransferase and aspartate ammonia-lyase, only a few AspDHs are characterized till date, and failure to reproduce the high activity of AspDH from *Rastonia eutropha* documented in the literature encouraged us to screen and characterize novel AspDHs from different origins. Interestingly, the AspDHs from *Klebsiella pneumoniae* 34618 (KpnAspDH) and *Delftia* sp. Cs1–4 (DelAspDH) showed successful soluble expression. KpnAspDH and DelAspDH containing C-terminal hexa-histidine tags were purified and characterized for their catalytic properties. Notably, in addition to its high reductive amination activity, DelAspDH exhibited considerable stability as compared to the other source of AspDHs. This work thus provides novel enzyme resource for engineering strains capable of producing AFAAs under anaerobic conditions.

## Introduction

Aspartate is the key metabolic node to synthesize a spectrum of amino acids in microbial fermentation processes, including threonine, lysine, isoleucine and methionine known as aspartate family amino acids (AFAA)^1^. AFAA has a diverse of applications ranging from feed to food and pharmaceutical industries, and therefore have important commercial values^1, 2^.

Three pathways exist in microbes directing the carbon flow away from TCA cycle towards the node of aspartate. The dominate pathway used in most industrial AFAA producing microbes (e.g. *Corynebacterium glutamicum* and *Escherichia coli*)^3–6^ was oxaloacetate (OAA) amination catalyzed by aspartate aminotransferase (EC:2.6.1.1) through the transfer of amino group from glutamate. OAA can also be converted to aspartate through the reductive TCA cycle where it is first reduced to malate and then fumarate before its conversion to aspartate by aspartate ammonia-lyase (EC:4.3.1.1). Another pathway, which is less studied, is the direct amination of OAA by aspartate dehydrogenase (AspDH, EC:1.4.1.21)^6^.

AspDHs catalyze the reversible conversion of oxaloacetate to aspartate with ammonium as the amino group donor. Perhaps owing to their low sequence and structural similarity compared with other members of amino acids dehydrogenase superfamily (e.g. glutamate dehydrogenase, alanine dehydrogenase, valine dehydrogenase), it was not until 2003 that the first AspDH was reported from a thermophilic bacterium, *Thermotoga maritima* ^7^. Since then, several AspDHs from other sources were characterized, including *Archaeoglobus fulgidus* ^8^, *Pseudomonas aeruginosa* ^9^, *Ralstonia eutropha* ^10^ and later two nitrogen-fixing bacteria *Rhodopseudomonas palustris* and *Bradyrhizobium japonicum* ^11^.

Currently, the main AFAA fermentation is aerobic process, where high energy input for aeration and cooling is required and a large portion of raw materials were lost as biomass and CO_2_. In this connection, anaerobic or microaerobic process is more economically appealing. OAA amination through aspartate aminotransferase may not be very efficient under anoxic conditions because a less active TCA cycle would not give forth to a sufficient glutamate/2-ketoglutarate pool^12^.

As such, AspDH, which utilizes ammonium as the amino donor, holds the promise for anaerobic production of AFAA. Unfortunately, the reservoir of available AspDHs for metabolic engineering purpose is very limited due to the relatively short research history of this enzyme. More importantly, in preliminary experiments, when testing the AspDH from *R. eutropha* (ReuAspDH, the one with the highest reported specific activity), only very low level of OAA amination activities could be detected, at least in our experimental system (seen in Result). In addition, a significant portion of ReuAspDH is in its insoluble form, which is an undesirable trait for metabolic engineering purpose because the formation of unfunctional insoluble aggregates would clearly be a waste of cellular resources. These issues motivate our search for better AspDHs for AFAA production.

In this study, we characterized several new AspDHs and successfully identified two AspDHs from *Klebsiella pneumoniae* 34618 and *Delftia* sp. Cs1-4 with high enzymatic activities. This work will provide a solid basis for developing strains capable of producing AFAA under anaerobic conditions.

## Results

### Evaluation of AspDHs from *R. eutropha* and *B. japonicum*

**Figure 1:**
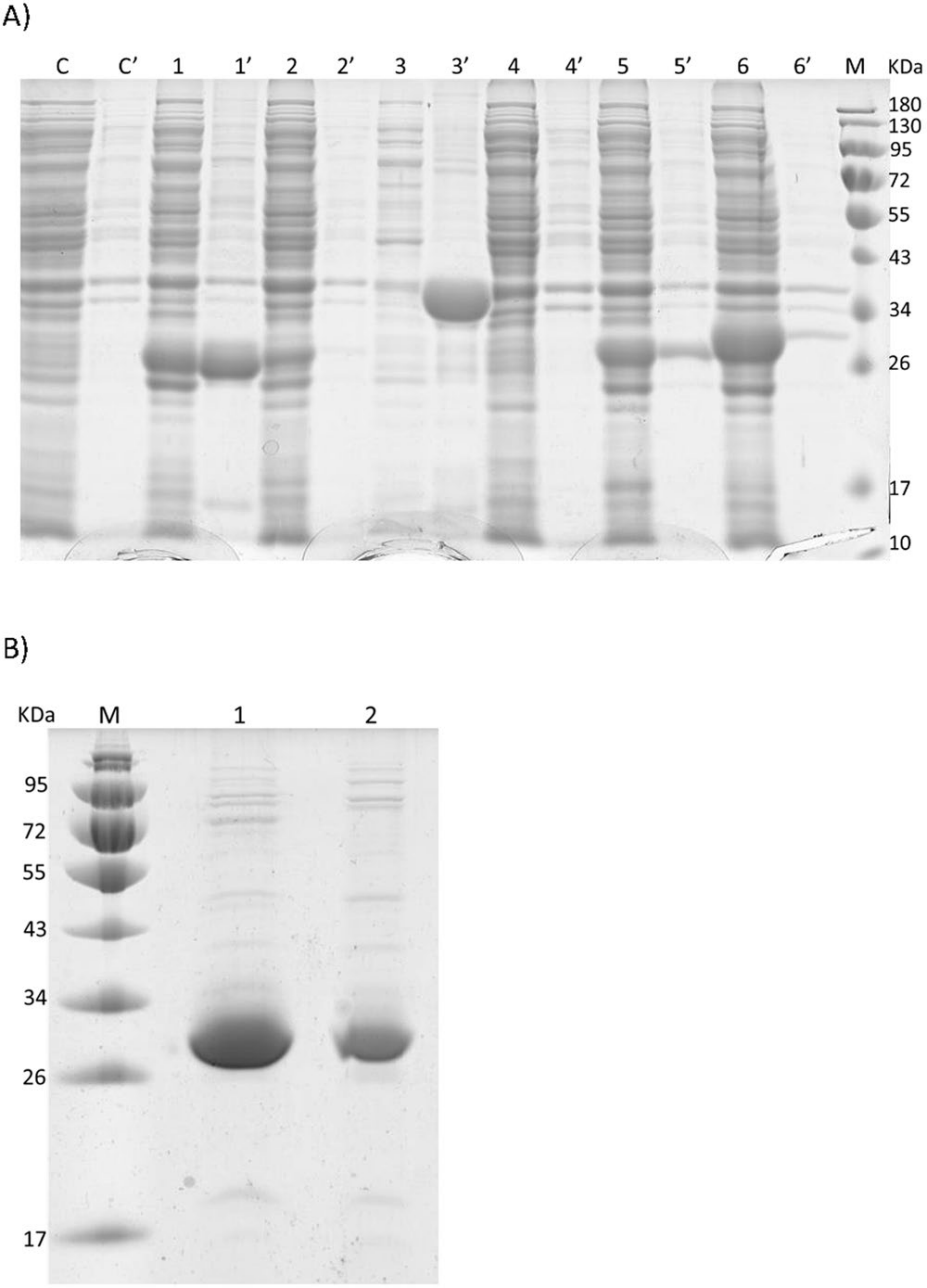
SDS-PAGE results of aspartate dehydrogenase expression and purification. (**A**) SDS-PAGE results of protein expression. M: molecular mass markers; Lane 1–6 and 1′–6′ represent the soluble and insoluble fractions of empty vector pED31, KpnAspDH, DelAspDH, MplAspDH, RedAspDH, ReuAspDH and BjaAspDH, respectively. (**B**) Purification of recombinant KpnAspDH and DelAspDH. M: molecular mass markers; Lane 1: KpnAspDH; Lane 2: DelAspDH.

To establish an efficient OAA amination system for AFAA fermentation, two AspDHs from *R. eutropha* (ReuAspDH) and *B. japonicum* (BjaAspDH), with very high reported catalytic activities were first evaluated in our system. The two genes were cloned into the pED31 vector under the control of P_LlacO1_ promoter, and transformed into in *E. coli* BL21(DE3). The recombinant strains were induced with 0.5 mM of IPTG at 30 °C for 12 h. SDS-PAGE analysis showed successful expression of the two enzymes, with a major fraction of proteins expressed in soluble form (Fig. 1A). For crude activity test using cell lysates, specific activities of 1.1 and 1.2 U/mg (pH8.0, 37 °C), were estimated for ReuAspDH and BjaAspDH respectively with NADPH as cofactor (Table 1). When NADH was used, ReuAspDH and BjaAspDH showed no difference in OAA amination (pH8.0, 37 °C) as compared with the empty vector control (data not show).

**Table 1:** The oligonucleotides used in this study.

Primers	Sequence 5′ → 3′
pED31-F	AGATCTGGGTACCTTAATTA
pED31-R	CATATGTATATCTCCTTCTTAA
Kpn-F	TTTAAGAAGGAGATATACATATGAAAAAAGTAATGCTGATTGGTTAC
Kpn-R	TAATTAAGGTACCCAGATCTTTAAGCCAGTTCGCGACAAG
Kpn-histag-R	TAATTAAGGTACCCAGATCTTTAGTGGTGGTGGTGGTGGTGAGCCAGTTCGCGACAAG
Del-F	CTTTAAGAAGGAGATATACATATGAACATCGCTGTAATCG
Del-R	TAGGTTAATTAAGGTACCCAGATCTTTAGATAGCGATTGCGGTAG
Del-histag-R	AGGTTAATTAAGGTACCCAGATCTTTAGTGGTGGTGGTGGTGGTGGATAGCGATTGCGGTAG
Mpl-F	CTTTAAGAAGGAGATATACATATGGTAATGGTTGGTATGC
Mpl-R	TAGGTTAATTAAGGTACCCAGATCTTTAAGTGCCCACAACGATC
Red-F	CTTTAAGAAGGAGATATACATATGCGCTACCAAGGGGTC
Red-R	TAGGTTAATTAAGGTACCCAGATCTTCAGATCACCACCGGTCTG
Bja-F	CTTTAAGAAGGAGATATACATATGACTGACCGTAAAGCATC
Bja-R	TAGGTTAATTAAGGTACCCAGATCTTTAAGTGCCTACGCACAG
Reu-F	TTTAAGAAGGAGATATACATATGTCTATGTTACATGTGAGCATG
Reu-R	TAATTAAGGTACCCAGATCTTTAGATGCTCACGGCATG

### Selection and evaluation of new AspDHs

The far lower than expected OAA amination efficiencies in previously reported AspDHs motivated us to search for new ones with better enzymatic characteristics. To increase the possibility of identifying novel AspDHs, we selected enzymes with relatively low identities with the already characterized ones. AspDHs from *Delftia* sp. Cs1-4 (DelAspDH) and *K. pneumon*iae 34618 (KpnAspDH) showed 65% and 42% of identity as compared with reported ReuAspDH, and therefore chosen for characterization in this work. In addition, two unconventional AspDHs from *Methanosphaerula palustris* (MplAspDH) and *Roseibacterium elongatum* (RedAspDH) were included, since they contain 302 and 126 amino acids, respectively while most of AspDHs contain 250 to 270 amino acids. Accordingly, four uncharacterized putative AspDHs, were chosen for cloning and expression in this work, using the same strategies applied for ReuAspDH and BjaAspDH.

SDS-PAGE results showed that of all AspDHs were successfully expressed except that from RedAspDH (Fig. 1A). Moreover, DelAspDH existed almost in soluble form while MplAspDH was expressed almost in insoluble form. For KpnAspDH, insoluble fraction was as much as the soluble one if not higher.

The crude activities of KpnAspDH and DelAspDH, were then evaluated for their aimination of OAA (Table 2). With NADH as cofactor, the crude specific enzyme activity DelAspDH was estimated to be 11.4 U/mg, while no activity could be detected for KpnAspDH. When NADPH was added, crude specific activities of 12.2 and 3.3 U/mg, were estimated significantly higher than those of ReuAspDH and BjaAspDH.

**Table 2:** Specific enzyme activities of recombinant AspDHs.

	Reu	Bja	Del	Kpn	Mpl	Red
Molecular mass (kDa)	27.9	28.6	27.2	26.8	31.3	13.4
Soluble expression	Y	Y	Y	Y	N	N
Enzyme activity (crude, amination, NADPH, U/mg)	1.2	1.1	12.2	3.3	—	—
Enzyme activity (purified, amination, U/mg)	—	—	32.8*	26.5**	—	—
Enzyme activity (purified, deamination, U/mg)	—	—	5.1	3.42	—	—

^*^OAA amination activity is determined with NADH as cofactor.

**OAA amination activity is determined with NADPH as cofactor.

### Purification of AspDH and activity tests

6xHis-tags were attached to the C-terminal of KpnAspDH and DelAspDH separately with Gibson assembly method, and expressed in *E. coli* BL21. Cell lysates were purified with Ni-affinity chromatography. Successful purification of the two enzymes was shown on SDS-PAGE analysis (Fig. 1B). For KpnAspDH, the highest specific activities were determined as 26.5 U/mg for the reduction of OAA and 3.42 U/mg for the oxidation of aspartate. For DelAspDH, the reduction and oxidation specific activities were 32.8 U/mg and 5.1 U/mg, respectively.

### Effects of pH and temperature on AspDH activity

**Figure 2:**
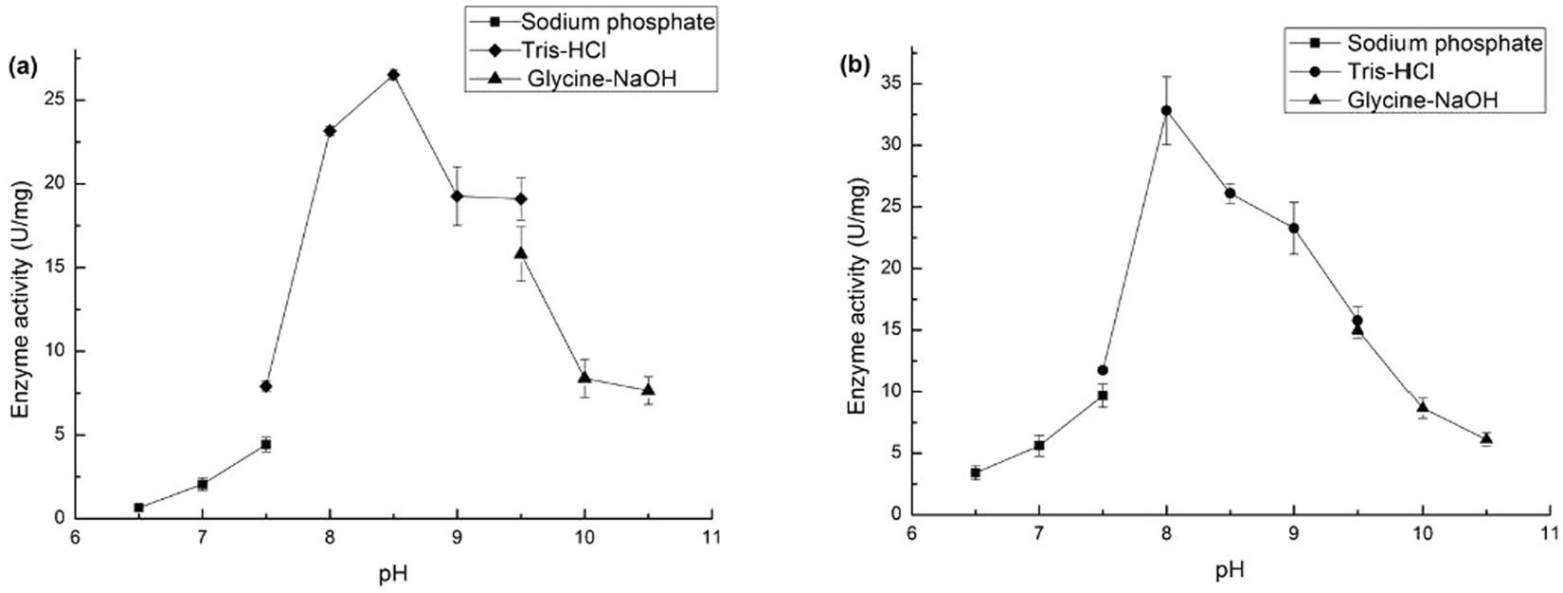
Characterization of the purified recombinant KpnAspDH and DelAspDH. (**a**) The optimal pH of KpnAspDH determined in various buffers from pH 6.5 to 10.5; the buffers used were glycine-NaOH (9.5–10.5) (black triangle), Tris-HCl (7.5–9.5) (black circle), Na_2_HPO_4_–NaH_2_PO_4_ (6.5–7.5) (black square). (**b**) The optimal pH of DelAspDH determined in the same buffer as above.

**Figure 3:**
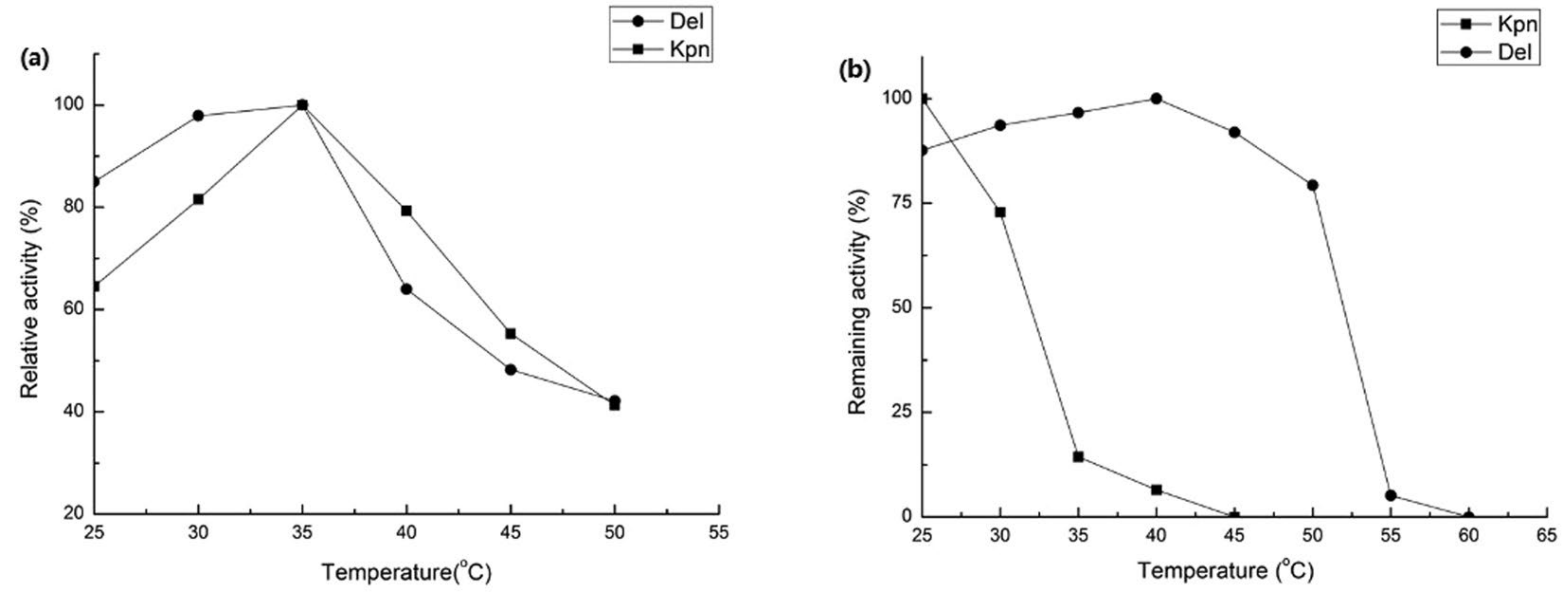
Characterization of the purified recombinant KpnAspDH and DelAspDH. (**a**) Optimal temperature measured in 100 mM Tris-HCl at pH 8.5 and 8.0, respectively. (**b**) Thermostability determined by measuring the residual activity after preincubation in temperatures ranging from 25 to 60 °C for 20 min.

The effect of pH and temperature on the two AspDHs were evaluated for both enzymes. Generally, both enzymes displayed significant activities under alkaline conditions (Fig. 2). The optimum pH for KpnAspDH and DelAspDH were of 8.5 and 8.0 respectively. In contrast, both enzyme displayed very little activity under neutral and acid environments. The optimum temperature of KpnAspDH and DelAspDH were both 35 °C (Fig. 3). The thermostability of the two enzymes were determined within the range of 25–60 °C for 20 min. KpnAspDH was rather unstable. Its specific activity decreased sharply with increased temperature and lost 75% activity even under its optimum temperature. We also observed serious aggregation of KpnAspDH when its concentration surpassed 2 g/L under 4 °C.

### Substrate specificity and kinetic parameters

To test whether pyruvate and 2-ketoglutarate can serve as alternative substrate for AspDH, the substrate specificities of KpnAspDH and DelAspDH were evaluated with the two metabolic intermediates as substrates. Results suggested that both enzymes showed high specificities with very low activity towards pyruvate and 2-ketoglutarate (Table 3). The kinetic parameters of the two enzymes were also determined. KpnAspDH generally showed preference to NADPH rather than NADH as predicted from the sequence analysis while DelAspDH showed higher activity with NADH (Table 4). In addition, both enzymes showed relatively low affinity towards ammonium.

**Table 3:** Substrate specificities of KpnAspDH and DelAspDH.

Substrate	KpnAspDH	DelAspDH
Reductive amination (%)		
Oxaloacetate	100	100
Pyruvate	3.9 ± 1.1	2.2 ± 0.3
2-ketoglutarate	3.5 ± 1.1	2.8 ± 0.5
Oxidative deamination (%)		
L-aspartate	100	100
L-glutamate	5.5 ± 1.8	3.9 ± 0.4
L-alanine	<1	1.5 ± 0.1

**Table 4:** Kinetic parameters of the KpnAspDH and DelAspDH.

	Substrate	*K*_m_(mM)	*k*_cat_(s^−1^)	*k*_cat_/*K*_m_(mM^−1^s^−1^)
KpnAspDH	NADH	0.146 ± 0.006	73.9 ± 2.1	505
	NADPH	0.080 ± 0.009	77.7 ± 3.0	960
	OAA(NADPH)	1.35 ± 0.17	69.0 ± 2.3	51.1
	NH_4_^+^(NADPH)	52.6 ± 4.5	75.5 ± 5.2	1.4
DelAspDH	NADH	0.069 ± 0.006	90.8 ± 1.9	1316
	NADPH	0.076 ± 0.006	86.3 ± 2.2	1136
	OAA(NADH)	1.24 ± 0.05	83.5 ± 1.7	67.3
	NH_4_^+^(NADH)	20.4 ± 2.0	89.1 ± 3.0	4.4

## Discussion

AspDHs have great potential to produce AFAA under anaerobic conditions. However, our results showed that the activities of previously reported AspDHs from *R. eutropha* and *B. japonicum* cannot suffice the need for efficient amination of OAA. Especially, ReuAspDH is reported to have a specific deamination activity of 137 U/mg, the highest of all previously reported AspDHs. Although its amination activity is not given, it is reasonable to expect the value to be much higher, based on studies on BjaAspDH and *R.palustris* AspDH as well as DelAspDH and KpnAspDH in this work, where amination activities of AspDHs were five-ten times higher than deamination activities. However, despite its large amount of expression, almost no amination activity for ReuAspDH was detected with cell lysates in our experimental system.

**Figure 4:**
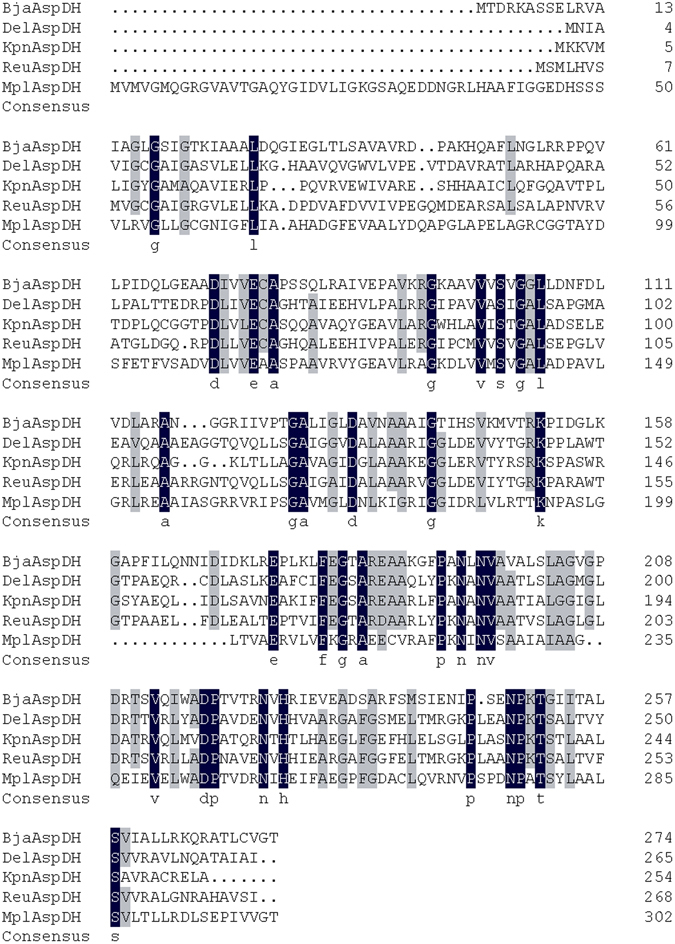
Multiple sequence alignments of aspartate dehydrogenases from *Bradyrhizobium japonicum* (BjaAspDH), *Delftia* sp. Cs1–4 (DelAspDH), *Klebsiella pneumoniae* 34618 (KpnAspDH), *Ralstonia eutropha* (ReuAspDH) and *Methanosphaerula palustris* (MplAspDH).

**Figure 5:**
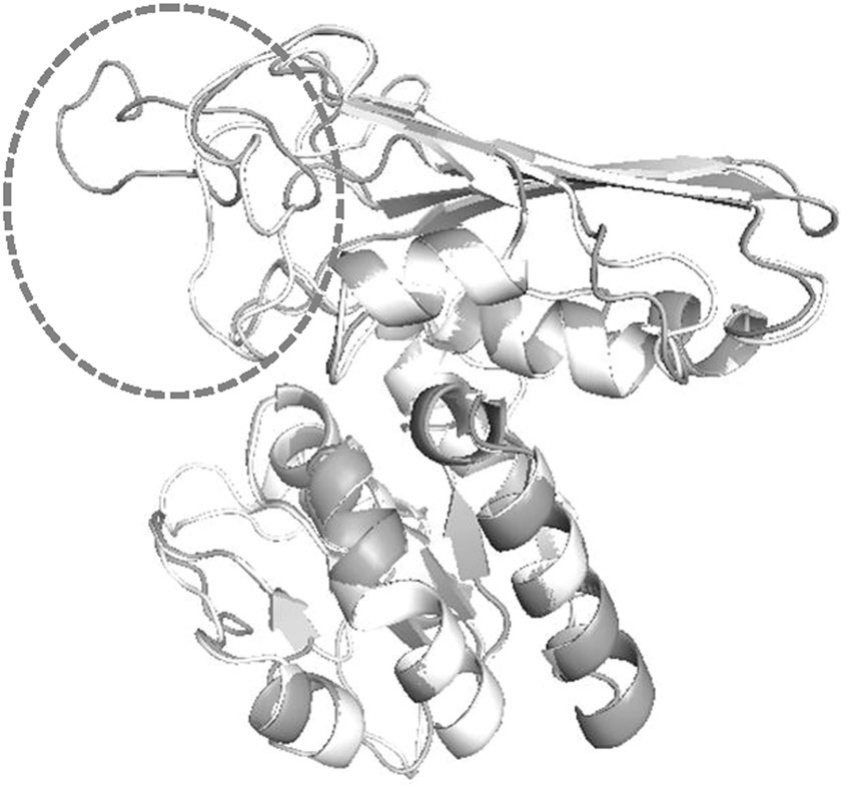
Structural alignment of KpnAspDH and DelAspDH. The structures of the two are shown in white and grey, respective. The position of the big loops is shown in dashed oval.

Failure to reproduce previous reports motivate our search for new AspDHs. Four new AspDHs from *K. pneumonia*, *Delftia* sp. Cs1–4, *M. palustris* and *R. elongatum* were evaluated for their expression and amination activities in this work. Structural analysis of *T. maritima* AspDH suggested AspDHs contain two discernable domains: N-terminal domain (residue 1–105) and C-terminal domain (residue 113–241)^7^. N-terminal domain contains a typical Rossmann fold, the protein motif which is predicted to bind coenzymes like NAD^+^, NADP^+^ or FAD. Sequence analysis showed that RedAspDH lacked N-terminal domain and thus the absence of activity in RedAspDH was not unexpected. Sequence comparison of the other five AspDH genes (Fig. 4) showed ReuAspDH, BjaAspDH and DelAspDH processed the canonical NADH/NAD^+^-binding motif (GxGxxG) at the start of N-terminal domain (Fig. 4). The GxGxxA variant in the corresponding position of KpnAspDH suggested its preference for NADPH/NADP^+^^13^ which was later confirmed by kinetic research. Structural models of KpnAspDH and DelAspDH were also established using *A. fulgidus* AspDH as template (PDB ID. 2DC1)^14^, and alignment of their models showed high similarity in overall structures (Fig. 5). The major difference between the two AspDHs was at the position of a big loop, where extending style was adopted for DelAspDH while flapping style was adopted for KpnAspDH.

There has been dispute on *K. pneumoniae* AspDH in previous literature. The AspDH from a *K. pneumoniae* strain IFO 13541 was been described with very low deamination activity (0.045 U/mg) by Okamura *et al*.^15^. However, Li and co-authors^16^ in their review have questioned the results because the analysis were obtained based on crude enzyme extracts and the reported molecular mass of 62 kDa is far larger than the typical weight of AspDHs (about 27 kDa). In addition, their bioinformatic analysis revealed that putative AspDHs with the expected molecular weight existed in some *K. pneumoniae* strains. Our work supported Li and co-authors’ argument by proving the catalytic function of the putative AspDH (254 AAs, 26.8 KDa) in *K. pneumonia*, which showed significantly higher specific deamination activity (3.42 U/mg) than the value reported by Okamura *et al*.

DelAspDH and KpnAspDH exhibited maximum OAA amination activity at pH 8.5 and 8.0 respectively, which was close to other characterized mesophilic AspDHs, such as PaeAspDH (pH 8.2), ReuAspDH (pH 8.2), BjaAspDH (pH 8–9) and RpaAspDH (pH 8–9). Moreover, the activities DelAspDH and KpnAspDH plummeted in pH range of 7.0–8.0. The trend was in accordance with the observations for BjaAspDH and RpaAspDH. However, BjaAspDH and RpaAspDH completely lost their activities at pH of 7.0–8.0 while DelAspDH and KpnAspDH can still maintain partial catalytic function. Especially for DelAspDH, a specific amination activity of 5.2 U/mg was detected at pH 7.0. This was of great importance for applications of AspDHs in AFAA fermentation, because physiological cytoplasmic pHs of most bacteria hosts were within the range of 7.4–7.7^17^.

The instability of some of the AspDHs has also been mentioned in literature. For instance, ReuAspDH was mildly unstable and tended to precipate from the solution even at 4 °C^10^. We found severe precipation of KpnAspDH when its concentration surpassed 2 g/L at 4 °C and its activity dropped sharply accordingly. In contrast, DelAspDH remained very stable and maintained most of its activity at 50°C. The reasons for this large difference in stability of the two AspDHs were unknown. The extending big loop in DelAspDH might hinder the surface contact between protein molecules, thus preventing its aggregation (Fig. 5).

## Materials and Methods

### Strains, plasmids and reagents

AspDH genes from *K. pneumoniae* 34618, *Delftia* sp. Cs1–4, *Methanosphaerula palustris*, *Roseibacterium elongatum*, *Bradyrhizobium japonicum* and *Ralstonia eutropha* JMP134 were synthesized by Genewiz (Suzhou, China) with codon optimization. *E. coli* BL21(DE3) pLysS (Transgen) was used for heterologous expression. The plasmid pED31 was used for the expression of AspDH. NAD^+^, NADH, NADPH, and NADP^+^ were purchased from Roche. All other chemicals used in this work were of analytical grade and commercially available.

### Construction of expression plasmid

*KpnAspDH, DelAspDH, MplAspDH, RedAspDH*, *BjaAspDH* and *ReuAspDH* were cloned into the vector pED31 at the *Nde*I and *Bgl*II sites, resulting in recombinant plasmids designated as pED31-KpnAspDH, pED31-DelAspDH, pED31-MplAspDH, pED31-RedAspDH and pED31-ReuAspDH, respectively. In addition, AspDH genes with a C-terminal His-tag were constructed for enzyme purification.

### Expression and purification of recombinant AspDH

The recombinant cells were grown at 37 °C in a 500 mL flask containing 100 mL LB medium with kanamycin (50 μg ml^−1^). Cells were induced at an OD_600_ of 0.6–0.8 with 0.5 mM IPTG and cultured at 30 °C for 12 h. Cells were harvested by centrifugation at 10,000 g for 5 min and resuspended in buffer 1 (50 mM sodium phosphate, pH 8.0; 300 mM NaCl; and 10 mM imidazole). Cells were broken by an ultrasonic cell disruptor and then debris were removed by centrifugation. The target proteins were bound to Ni^2+^ affinity chromatography and eluted in a buffer 2 (50 mM sodium phosphate, pH 8.0; 300 mM NaCl; and 250 mM imidazole). The target enzyme expression was detected by SDS-PAGE and the protein concentration was measured by Bradford method.

### Enzyme activity assays

The reduction reaction mixture (200 μL) contained 4 mM OAA, 0.2 mM NAD(P)H, 100 mM Tris–HCl buffer (pH 8.2), 100 mM NH_4_Cl. The oxidation reaction was determined by mixtures containing 50 mM L-Aspartate, 2.5 mM NAD(P)^+^ in 100 mM glycine-NaOH buffer (pH 10). The activity was determined by measuring the increase or decrease of NAD(P)H at 340 nm (extinction coefficient ε = 6.22 mM^−1^ cm^−1^). All reactions were initiated by adding enzyme and performed in 96-well plate reader. One unit of activity was defined as the amount of enzyme required to reduce or oxidize 1 μmol of NAD(P)^+^ or NAD(P)H per min.

### Effects of pH and temperature on enzyme activity and temperature stability

The optimum pH was determined using following buffers: 100 mM sodium phosphate (pH 6.5–7.5), 100 mM Tris-HCl (pH 7.5 to 9.5), and 100 mM glycine-NaOH (pH 9.5–10.5). The optimum temperature was determined in different temperatures ranging from 25 to 50 °C. The temperature stability was determined by incubating enzymes in 100 mM sodium phosphate (pH 7) at temperatures ranging from 25 to 60 °C for 20 min.

### Substrate specificity and kinetic parameters

The substrate specificity was measured under following system: 8 mM pyruvate or 2-ketoglutarate, 0.2 mM NAD(P)H, 100 mM Tris–HCl buffer (pH 8.2), 100 mM NH_4_Cl for amination reaction, 50 mM L-Glutamate or L-Alanine, 2.5 mM NAD(P)^+^, 100 mM glycine-NaOH buffer (pH 10) for oxidation reaction. The *K*_m_ and *k*_cat_ values were determined by changing different concentrations of one substrate while keeping concentrations of the other substrates saturated.

### Sequence analysis and structure modelling

Sequence analysis was performed using DNAMAN 6.0 software. Structural models of KpnAspDH and DelAspDH was built in SWISS-MODEL program using *A. fulgidus* AspDH as template (PDB ID. 2DC1). Sequence alignment was performed using Pymol (http://www.pymol.org).
